# Ultrahigh relaxivity and safe probes of manganese oxide nanoparticles for *in vivo* imaging

**DOI:** 10.1038/srep03424

**Published:** 2013-12-05

**Authors:** J. Xiao, X. M. Tian, C. Yang, P. Liu, N. Q. Luo, Y. Liang, H. B. Li, D. H. Chen, C. X. Wang, L. Li, G. W. Yang

**Affiliations:** 1 State Key Laboratory of Optoelectronic Materials and Technologies, Institute of Optoelectronic and Functional Composite Materials, Nanotechnology Research Center, School of Physics & Engineering, Sun Yat-sen University, Guangzhou 510275, Guangdong, P. R. China; 2 State Key Laboratory of Oncology in South China, Imaging Diagnosis and Interventional Center, Sun Yat-sen University Cancer Center, Guangzhou 510060, P. R. China; 3 Department of Biomedical Engineering, Guangzhou Medical University, Guangzhou 510182, P. R. China; 4 These authors contributed equally to this work.

## Abstract

Mn-based nanoparticles (NPs) have emerged as new class of probes for magnetic resonance imaging due to the impressive contrast ability. However, the reported Mn-based NPs possess low relaxivity and there are no immunotoxicity data regarding Mn-based NPs as contrast agents. Here, we demonstrate the ultrahigh relaxivity of water protons of 8.26 mM^−1^s^−1^ from the Mn_3_O_4_ NPs synthesized by a simple and green technique, which is twice higher than that of commercial gadolinium (Gd)-based contrast agents (4.11 mM^−1^s^−1^) and the highest value reported to date for Mn-based NPs. We for the first time demonstrate these Mn_3_O_4_ NPs biocompatibilities both *in vitro* and *in vivo* are satisfactory based on systematical studies of the intrinsic toxicity including cell viability of human nasopharyngeal carcinoma cells, normal nasopharyngeal epithelium, apoptosis in cells and *in vivo* immunotoxicity. These findings pave the way for the practical clinical diagnosis of Mn based NPs as safe probes for *in vivo* imaging.

Magnetic resonance imaging (MRI) is a routine diagnostic tool in modern clinical medicine. One of significant advantages of MRI is able to obtain three-dimensional tomographic information about anatomical details with high spatial resolution and soft tissue contrast in a non-invasive and real-time manner^1^,^2^,^3^,^4^,^5^. In order to compensate the innate low sensitivity, the positive or T_1_ contrast agents are employed to increase contrast between organs of interest and normal organs by accelerating the longitudinal relaxivity (r_1_) of water protons, which leading to a brightening of MR image^6^,^7^,^8^. The majority of T_1_ MRI contrast probes are currently based on gadolinium (Gd^3+^) in the form of paramagnetic chelates^9^,^10^,^11^. However, their uses are occasionally associated with nephrogenic system fibrosis (NSF), which suggests a need of finding alternatives^12^,^13^,^14^. Recently, nanoparticles (NPs) have been extensively used in biomedical application^15^,^16^,^17^,^18^,^19^. As MRI contrast agents, NPs with high relaxivity and low toxicity are most expected. Among all the candidates, Mn-based NPs are regarded as promising alternatives due to their lower intrinsic toxicity than that of Gd^3+^ and increasing attention in neuroscience research^20^,^21^,^22^. However, the development of Mn-based NPs is hindered by two bottlenecks. One is that the Mn-based NPs with high relaxivity have not been still achieved, e.g., the relaxivity of the reported Mn-based NPs is usually lower than that of the commercial Gd-based agents (4.11 mM^−1^s^−1^)^23^,^24^,^25^. Another is that there have not been any pre-clinical reports on *in vitro* and *in vivo* studies of toxicity of Mn-based NPs^20^,^21^,^22^,^23^,^24^,^25^. Nanotoxicity^26^, especially immunotoxicity^27^,^28^,^29^, has emerged as one of the critical issues to make NPs into practical clinical applications. Although the standardized assessments on immunotoxicity of NPs in biomedical products have not yet been established, it's essential to assess the immune response to the nanoparticles in the pre-clinical research^30^,^31^.

Here we synthesize the ligand-free Mn_3_O_4_ NPs by a simple and green laser-based technique, i.e., laser ablation in liquid (LAL)^32^,^33^,^34^,^35^,^36^,^37^,^38^. Our measurements indicate that the water proton relaxivity is 8.26 mM^−1^s^−1^ when adding the Mn_3_O_4_ NPs, which is twice higher than that of the commercial Gd-DTPA contrast agent (4.11 mM^−1^s^−1^) and the highest value reported to date for Mn-based NPs^23^,^24^,^25^. We also for the first time take systematically the *in vitro* and *in vivo* pre-clinical studies on the toxicity of the as-synthesized Mn_3_O_4_ NPs and the pharmacokinetics assays. All the measurements confirm that this Mn-based nanoprobe is safe in biocompatible due to lack of any potential toxicity. Therefore, these results demonstrate that the LAL-derived Mn_3_O_4_ NPs are strong candidates as effective and safe targeted probes for early tumor diagnosis, and superior to the commercial Gd-based contrast agents in terms of contrast enhancement with a satisfactory biocompatibility.

## Results

### Structure, morphology and component of Mn_3_O_4_ NPs

**Figure 1:**
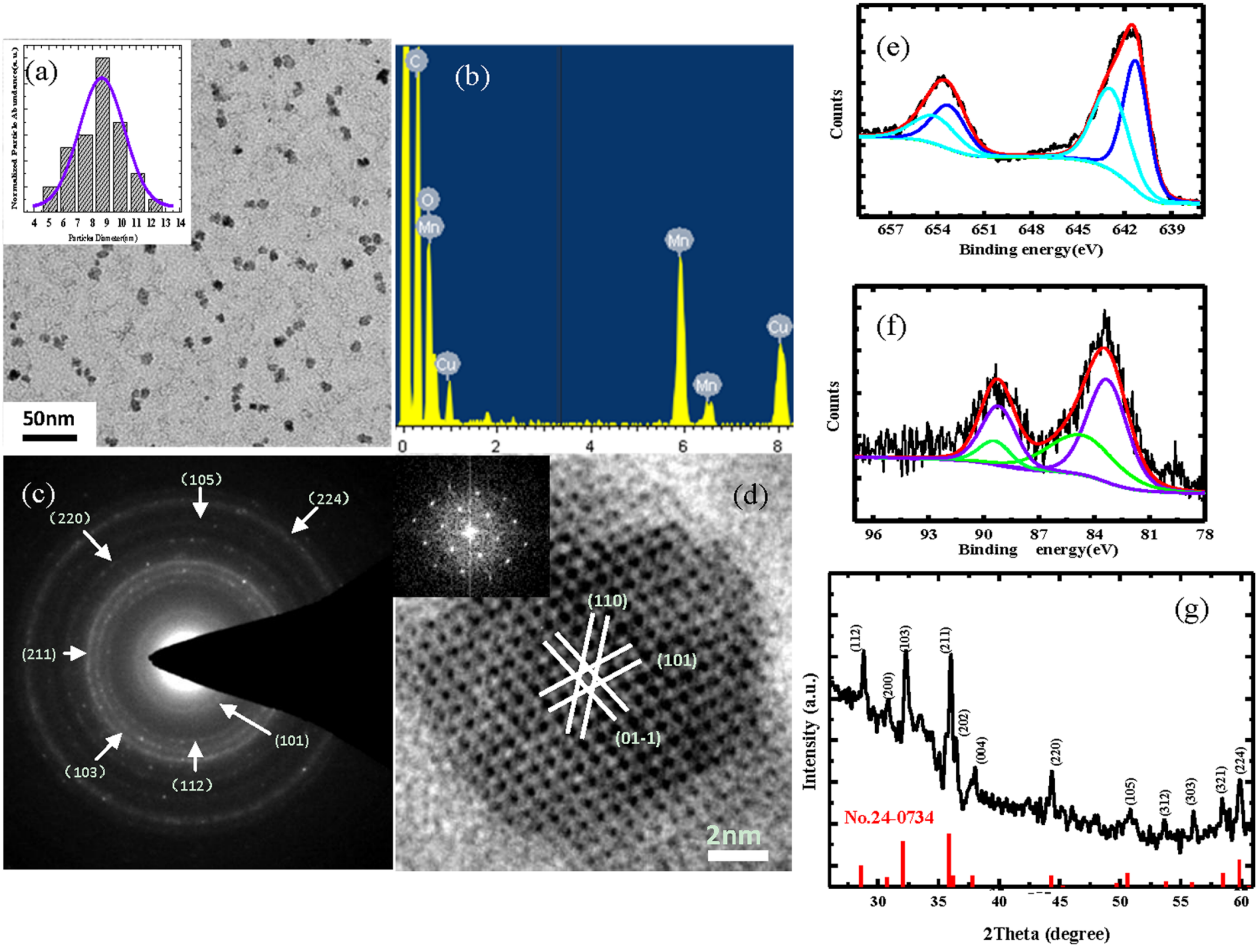
Characterizations of structure, morphology and component of Mn_3_O_4_ NPs. (a) TEM image of dispersive Mn_3_O_4_ NPs. The distribution histogram and its Gaussian fitting curve (inset) to demonstrate that the mean size of the sample is about 9nm. (b) EDS spectrum of Mn_3_O_4_ NPs. (c and d) The corresponding selected-area electron diffraction pattern and high resolution TEM image, the inset in (d) shows a fast Fourier transform analysis of individual Mn_3_O_4_ NPs. (e and f) XPS spectrum of Mn2p and Mn3s level. (g) XRD pattern of the as-synthesized Mn_3_O_4_ NPs.

From Fig. 1a, we can see the uniform and dispersive NPs with a diameter of about 9 nm (calculated from about 200 nanoparticles). The energy dispersive spectrum (Fig. 1b) shows the products are composed of Mn and O elements, and the Cu and C peaks originate from the copper grid and amorphous carbon film support, respectively. The selected area electronic diffraction (Fig. 1c) reveals that these NPs are consistent with strong ring patterns of the tetragonal Mn_3_O_4_ structure. The high resolution TEM (Fig. 1d) also confirms this result. The XPS measurements are employed to analyze the Mn oxidation states so as to determine which chemical valence state is responsible for shortening relaxation time. From Fig. 1e, the binding energy of Mn 2p_3/2_ peaks components are 641.2 and 642.9 eV, which correspond exactly with the data reported respectively for Mn^2+^ and Mn^4+^ 39,40,41. The splitting of the Mn 3s doublets (Fig. 1f) are 5.8 and 4.6 eV, which are in agreement with the relative value of Mn^2+^ and Mn^4+^ valence states^39^,^40^,^41^. Therefore, XPS analyses show that the external layers of the products are consisted of Mn^2+^ and Mn^4+^. Note that Mn^2+^ has 5 unpaired electrons, which are more than other valence states of Mn ion. Fig. 1g shows the XRD pattern of the products. Clearly, all the peaks are indexed to Mn_3_O_4_ with the tetragonal structure (JCPDS no.24-0734) without metallic manganese or other oxide phase, which indicates that the as-synthesized Mn_3_O_4_ NPs are crystalline and of high purity.

### *In vitro* and *in vivo* MR imaging

**Figure 2:**
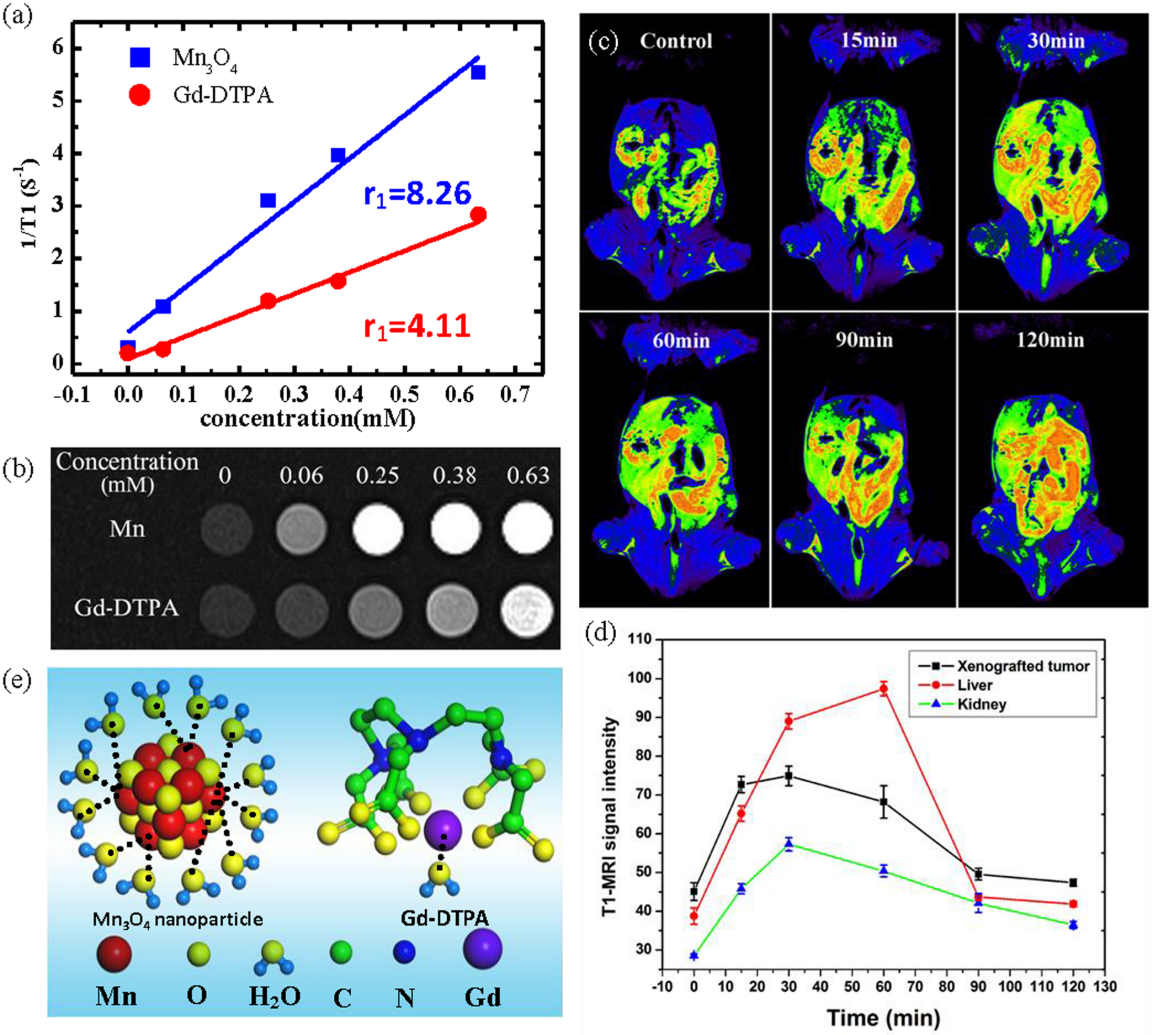
*In vitro* and *in vivo* MR imaging. (a) The relaxivity (r_1_) of Mn_3_O_4_ NPs and commercially Gd-DTPA detected by nuclear magnetic relaxation dispersion (NMRD). (b) T_1_-weighted phantom MRI of various concentrations of Mn_3_O_4_ NPs (upper row) and Gd-DTPA (lower row). (c) Representative dynamic contrast-enhanced T_1_-weighted MRI of a nasopharyngeal carcinoma (NPC) CNE-2 xenografted tumor (white arrow), liver and kidney in Balb/c nude mice obtained at 0, 15, 30, 60, 90, 120 min, respectively, after intravenous administration of Mn_3_O_4_ NPs (15 μmolkg^−1^). (d) Dynamic enhancement curve of xenografted tumor, liver and kidney. (e) Schematic illustration of interaction between contrast agent (Mn_3_O_4_ NPs (left side) and Gd-DTPA (right side)) and water.

The MRI properties of the Mn_3_O_4_ NPs in water are characterized by a 3T MR scanner. The molar relaxivity (Fig. 2a) is obtained by measuring the relaxation rate of water protons with increasing concentrations of NPs, and is calculated to be 8.26 mM^−1^s^−1^. This value is twice higher than that of Gd-DTPA (4.11 mM^−1^s^−1^) and the highest value reported to date for Mn-based NPs (Table 1)^23^,^24^,^42^,^43^,^44^,^45^,^46^,^47^. These nanocrystals are also tested in PBS solution in order to simulate the culture medium. The relaxivity is calculated to be 6.79 mM^−1^s^−1^ (shown in Supplementary Fig. S2a). Meanwhile, the Mn_3_O_4_ NPs provide improved contrast enhancement compared to Gd-DTPA contrast agents from Fig. 2b.

To assess the *in vivo* MR imaging, the Mn_3_O_4_ NPs are intravenously administrated into Balb/c nude mice with nasopharyngeal carcinoma (NPC) CNE-2 xenografted tumors. Dynamic contrast enhanced T_1_-weighted MRI of liver, kidney and xenografted tumor is obtained. As shown in Fig. 2c and d, the T_1_-weighted MR images clearly show a high contrast enhancement of the xenografted tumor (white arrow) after injecting the Mn_3_O_4_ NPs at 30 min. In addition, the corresponding kidney enhancement and grey-scale image are shown in Supplementary Fig. S3 and S4. Note that the administered concentration of Mn in our MRI assessment is 15 μmolkg^−1^, which is only 1/7-1/14 of standard clinical dose of Gd-DTPA (0.1–0.2 mmolkg^−1^)^48^. The same dose of Gd-DTPA is also injected (shown in Supplementary Fig. S5), the signal enhancement is about 23%, which is lower than that of as-synthesized Mn contrast agent (64%). Therefore, both the *in vitro* and *in vivo* investigations confirm that the Mn_3_O_4_ NPs are more effective than Gd-DTPA in T_1_-weighted images.

The longitudinal relaxivity is proportional to the hydration number of water (q) that coordinates to the unpaired electrons of contrast agents^9^. Referring to the commercially available clinical contrast agent Gd-DTPA, the ligand DTPA forms a sufficiently stable complex around the Gd^3+^ ion, and only one coordination site is open up for water ligation, however, the Mn^2+^ carries five unpaired electrons, which offer more free sites for water ligation and result in higher r_1_^9^,^49^. Fig. 2e provides a schematic illustration of interaction between contrast agent (Mn_3_O_4_ NPs and Gd-DTPA) and water.

### Evaluation of toxicity *in vitro* and *in vivo*

**Figure 3:**
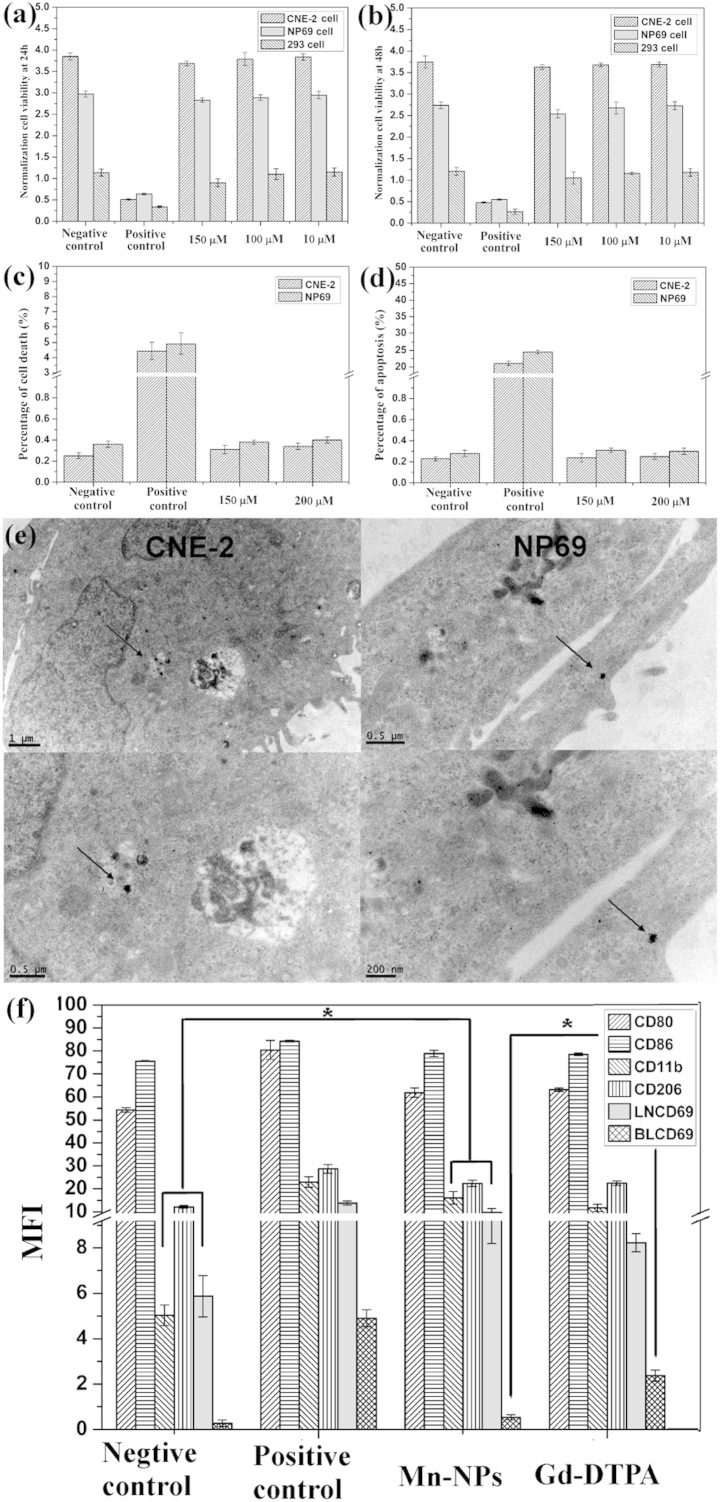
Toxicity assay of Mn_3_O_4_ NPs *in vitro* and *in vivo*. (a–b) The data of cell viability on CNE-2, NP69 and 293 cells incubated with different concentrations (150 μM, 100 μM, and 10 μM) of the Mn_3_O_4_ NPs for 24 and 48 h. (c–d) Cell death and Apoptosis rate of CNE-2 and NP69 cells were measured by flow cytometry at 48 h after incubation of PBS, LPS, Mn_3_O_4_ NPs (150 μM and 200 μM). Cells were stained by annexin V and PI. (e) Cells absorption data of the Mn_3_O_4_ NPs. TEM images of CNE-2 and NP69 at 12 h after incubation with the Mn_3_O_4_ NPs (100 mmol/L). (f) Immunotoxicity assay *in vivo*. CD80, CD86, CD11b and CD206 expression of monocytes/macrophages in peripheral blood, as well as CD69 cytokine of adaptive immune in lymphocyte cells of peripheral blood (BL) and lymph nodes (LN). * P < 0.05 compared with Gd-DTPA group.

To evaluate the toxicity of the Mn_3_O_4_ NPs *in vitro*, cell viability of L929 cells, 293 cells, NP69 cells (normal nasopharyngeal epithelium) and CNE-2 (human nasopharyngeal carcinoma) cells is determined by [3-4,5-dimethyl thiazol-2-yl]-2,5-diphenyltetrazolium bromide succinate (MTT) assay at 24 and 48 h, respectively. Clearly, the Mn_3_O_4_ NPs do not significantly affect cell viability in Fig. 3a, b Supplementary Fig. S6, and the cytotoxicity of the Mn_3_O_4_ NPs is very negligible. In addition, death and apoptosis of NP69 cells and CNE-2 cells are evaluated by flow cytometry stained with Annexin-V/PI. Fig. 3c, d and Supplementary Fig. S7 confirm the results of MTT assay. Moreover, as shown in Fig. 3e, TEM images of CNE-2 cells and NP69 cells show that the nanoprobes are absorbed by cells at 24 h. These results thus demonstrate that our nanoprobes have no effects on cells survival.

To further investigate the toxicity of the Mn_3_O_4_ NPs *in vivo*, the immunotoxicity are evaluated in Balb/c mice. In brief, we determine the typical cytokines of innate immune including CD206, CD11b, and CD80/CD86 of monocytes/macrophages in peripheral blood, as well as CD69 cytokine of adaptive immune in lymphocyte cells of peripheral blood and lymph nodes. The results are showed in Fig. 3f and Supplementary Fig. S8. There is significant difference between NPs and positive control groups (LPS), which indicating that the measurement is credible. Though there is statistical difference between Mn_3_O_4_ NPs and the negative control groups (PBS) on the expression levels of CD11b, CD206 and LNCD69, which indicates that our nanoprobes do slightly stimulate the immune response system, no obvious difference is found between the Mn_3_O_4_ NPs and Gd-DTPA groups. Besides, the blood CD69 of the Mn_3_O_4_ NPs group is decreased slightly compared to that of the Gd-DTPA group, which confirming that the as-synthesized Mn-based NPs are as safe as Gd-DTPA. Because Gd-DTPA is the commercial and widely used clinical contrast agent, Mn_3_O_4_ NPs might exhibit a little immunotoxicity, but the immune response can be acceptable by body *in vivo*.

### Pharmacokinetics assays including half-time, biodistribution, and excretion

**Figure 4:**
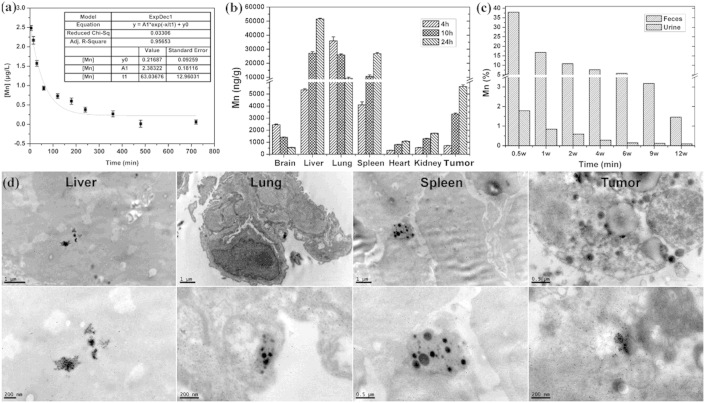
Pharmacokinetic characterizations of Mn_3_O_4_ NPs. (a) Half-life in the blood is determined by ICP-MS, and regularly measured the concentrations of Mn in blood samples (*n* = 3). (b) Concentrations of Gd were quantified in the brain, liver, lung, spleen, heart, kidney, and the tumor tissue (*n* = 3) at 4, 10 and 24 h, respectively, after intravenous injection (15 *μ*mol/kg). (c) Excretion of Mn is assayed in feces and urine of mice every week (*n* = 3) up to 12 weeks. (d) The biodistribution at the subcellular lever. TEM images of liver, lung, spleen and xenografted tumors in nude mice at 4 h after intravenous administration of the Mn_3_O_4_ NPs (15 *μ*mol/kg).

Assessing the toxicity of nanobased biomedicine is involved with physicochemical characteristics. Thus, we first measure the stability of our nanoprobes in blood. The half-life of the Mn_3_O_4_ NPs is 63.04 (±12.96) min in blood (Fig. 4a), which is much longer than that of Gd-DTPA (20 min)^48^. The longer half-life shows the favorable stability and low blood toxicity *in vivo*. Importantly, it can effectively improve the accumulation of nanoprobes in tumor tissue during circulation and the sensitivity of MR imaging.

To further investigate the biodistribution and excretion of the Mn_3_O_4_ NPs, the quantitative analysis on Mn concentration is measured by inductively coupled plasma mass spectrometry (ICP-MS) in typical organs, xenografted tumor tissues, feces and urine of mice. From Fig. 4b, we can see that our nanoprobes accumulate gradually in the lung, liver, spleen, and tumor tissue, but few are found in the brain, heart, and kidney. The exact concentrations of Mn in different organs are listed in the Supplementary Table S1. Interestingly, the Mn_3_O_4_ NPs accumulate increasingly in tumor tissues via the repeated blood circulation, which suggesting that it is a potential tumor-targeting nanoprobe. Moreover, as shown in Fig. 4c, about 50% of Mn is excreted via the hepatobiliary transport system within 1.5 weeks. Though hepatobiliary excretion is a slow process, it can still effectively decrease the occurrence of toxicity due to the accumulation of NPs. Importantly, the biodistribution at the subcellular level is observed by TEM, Fig. 4d shows our nanoprobes are mainly localized in the macrophages in the liver, lung, and spleen, as well as in the cytoplasm of epithelial cells in the xenografted tumor tissue. Since the as-synthesized NPs are dispersed inside the tissues with little aggregation, which leading to gradual excretion and minimal cell toxicity. In addition, no abnormalities are found in histological sections of the main organ including brain, heart, kidney, liver, lung, and spleen (Supplementary Fig. S9), which suggest that the cellular integrity and tissue morphology are not affected by our nanoprobes.

## Discussion

The reason that the r_1_ value of the Mn_3_O_4_ NP synthesized by LAL is higher than that of other Mn-based NPs is still unclear. We suggest that the distance between water and nanoprobes can be one of the influence factors. The T_1_ relaxation of water protons is affected by Mn ion via dipolar mechanism, which is a multifaceted phenomenon. Water in close proximity to ion is relaxed and paramagnetic T_1_ relaxation enhancement is a spin-lattice effect, which requires a direct contact between surface Mn ion and water^9^,^10^,^39^. Based on the Solomon-Bloembergen-Morgan (SBM) theory^50^,^51^,^52^,^53^, a classical existing theory of interpreting relaxation of water protons in the present of contrast agent, the relaxivity has a 1/d^6^ dependence on the distance (d) between contrast agents and water proton, which can be simplified as: r_1_∝d^−6^. So, in this case, the shorter the distance between external Mn ion and water proton is, the higher relaxivity is. Additionally, the surface of the LAL-derived NPs is not blocked by any chemical ligands or residues of any reducing agents, which reduce the distance between Mn ion and water proton. This hypothesis has been verified by changing deionized water into 5 mM SDS solution when ablating the target. The FTIR spectrum exhibits that SDS has coated the surface of Mn_3_O_4_ nanocrystals^54^,^55^, the corresponding relaxivity is dropped to be 1.75 mM^−1^s^−1^ (shown in Supplementary Fig. S3b–3c), which is much lower than the relaxivity of products synthesized in deionzed water (8.26 mM^−1^s^−1^). Therefore, clean surface remains when LAL in deionized water, which is likely to result in higher r_1_.

In summary, we have synthesized the Mn_3_O_4_ NPs with the ultrahigh relaxivity of 8.26 mM^−1^s^−1^ by a simple and green laser-based technique. We further demonstrate that these Mn-based NPs are safe and effective targeted probes for *in vivo* imaging based on the *in vitro* and *in vivo* assessments of biocompatibility, especially the evidence of immunotoxicity. These findings break through the bottleneck in the application of Mn-based NPs for MRI and pave the way for the practical clinical diagnosis of Mn-based NPs as safe probes for *in vivo* imaging.

## Methods

We stated that all the experiments have been approved by the State Key Laboratory of Oncology in South China of China in this study.

### Mn_3_O_4_ NPs synthesis

The details of laser ablation in liquids have been reported in our previous works^33^,^34^. In this case, a manganese target (99.99% purity) is firstly fixed on the bottom, and then the deionized water is poured into the chamber until the target in covered by 8 mm. Then, a second harmonic produced by a Q-switch Nd: YAG laser device with a wavelength of 10 Hz, and laser pulse power of 70 mJ, is focused onto the surface of manganese target. The spot sized is 1 mm in diameter and the whole ablation lasts for 30 min. The experimental setup is Supplementary shown in Fig. S1. As a result, the brown colloid solution is synthesized and collected into a cuvette. After 24 hours, the upper clear liquid is collected for further measurement.

### Products characterization

X-ray diffraction (XRD) was performed with a Rigaku D/Max-IIIA X-ray diffractometer with Cu Kα radiation (λ = 1.54056 Å, 40 kV, 20 mA) at a scanning rate of 1° s^−1^, and transmission electron microscopy (TEM) was carried out with a JEOL JEM-2010HR instrument at an accelerating voltage of 200 kV, equipped with an energy-dispersive X-ray spectrometer (EDS). Sample was ultrasounded for a few minutes and then one drop pipette onto a carbon support film on a copper grid. These techniques are used to identify the structure and morphology of as-synthesized samples. XPS (ESCAlab250) is employed to analyze the composition of the surface of samples. Inductively coupled plasma-atomic (ICP) emission spectrometry using a ThermoFisher iCAP6500Duo has been employed to analyze the concentration of Mn, with an incident power of 1150 W, a plasma gas flow of 14 L/min, and an atomization gas flow of 0.6 L/min.

### MRI *in vitro*

Various samples of Mn concentrations (from 0.06 to 0.63 mM) are in 1.5 ml EP tubes, and subject to T_1_-weighted phantom MRI by 3.0 T clinical scanner (Siemens Medical Solutions, Erlangen, Germany). The concentration of Mn is obtained by inductively coupled plasma atomic emission spectroscopy (ICP-AES, Spectro ciros vision, Spectro, Germany). The sequences are TSE T_1_ axial (5% dist. Factor, slice thickness 2.0 mm, FOV 64 mm, TE 12 ms, TR 600 ms, six averages). All data are analyzed by picture archiving and communications system (PACS).

### MRI *in vivo*

Balb/c nude mice with NPC CNE2 xenografted tomrs are induced anesthesia by intraperitoneal injection of 0.1 mebumalnatrium (10 μL per g weight), than injected with 15 μmol/kg of the Mn_3_O_4_ NPs by the tail vein, scanned on a 3.0 T Siemens Trio MRI scanner (Siemens Medical Solutions, Erlangen, Germany) using a surface coil with 3 inch in diameter. The control group is the uninjected mice. T_1_-weighted images are obtained at 0, 15, 30, 60, 90 and 120 min after intravenous administration in the axial orientations. The sequences are the same as the MRI *in vitro*. To be not biased toward aberrantly enhanced regions, the entire tumor is generated the normalized histograms of signal intensity.

### Cytotoxicity assay

The Human embryonic kidney (HEK) 293 cells in logarithmic growth period are incubated with different concentrations of the Mn_3_O_4_ NPs (150 μM, 100 μM, and 10 μM) in Dulbecco's modified Eagle's medium (DMEM)/F12 in 96-well plates, at 37°C, 5% CO_2_, treated only with culture media as negative control, treated with 0.5% dimethyl sulfoxide (DMSO) as positive control, all groups are cultured for 24 and 48 h post-treatment, respectively. Then, added 20 μL of MTT for another 4 h of incubation, replaced culture media with 100 μL DMSO for 10 min. The samples are measured by a microplate reader (Bio-Rad, USA) at 490 nm.

### Apoptosis assay

The NP69 cells and CNE-2 cells in 6-well plants are incubated with PBS (negative control), LPS (positive control) and the Mn_3_O_4_ NPs (150 μM and 200 μM) for 48 h, washed twice in cold PBS (phosphate-buffered saline) by gentle shaking, then resuspended cell pellet with 200 μL Binding Buffer (1×) at 4 × 10^5^ cells/ml, added 5 μL Annexin V-FITC (eBioscience) into 195 μL cell suspension, mixed and incubated for 10 min at room temperature, washed cells twice in 200 μL Binding Buffer (1×), and resuspended in 190 μL Binding buffer (1×), then added 10 μL PI (Propidium Iodide) (20 μg/mL), the samples are measured on a FACScan (Becton Dickinson, Mountain View, CA).

### Immunotoxicity assay *in vivo*

Male Balb/c mice are 6–8 weeks old, 20 mice are divided into 4 groups at random: PBS (100 μL, Negative control), Gd-DTPA (15 μmol/kg), LPS (100 μL, Positive control), the Mn_3_O_4_ NPs (15 μmol/kg). Peripheral blood or lymphocytes are measured after tail vein administration at 48 h by flow cytometry, stained with anti-mouse CD3-PE, anti-mouse CD11b-FITC, anti-mouse CD80/CD86-PE, anti-mouse CD69-FITC (Becton Dickinson PharMingen), and anti-mouse F4/80 antigen APC, anti-mouse CD206-PE (eBioscience).

### Pharmacokinetic characterizations

Concentrations of Mn are measured by ICP-MS (Thermo Instrument System Inc. USA) for all the samples of pharmacokinetics.

#### Half-life in the blood

The half-life in the blood is determined by 30 clean Kunming white mice (50% males and 50% females). Blood is obtained by the tail veins at 5, 15, 30, 60, 120, 180, 240, 360, 480, and 720 min, respectively, after tail vein administration of the Mn_3_O_4_ NPs (15 *μ*mol/kg).

#### Biodistribution at the organ and subcellular level

At the organ lever, brain, liver, lung, spleen, heart, kidney, and tumor are collected at 4, 10, and 24 h, respectively, after nanoprobes injection (15 *μ*mol/kg). At the subcellular level, liver, lung, spleen, and tumor are obtained at 4 h after injection. Samples were measured by TEM.

#### Excretion of the nanoprobes

Feces and urine of mice are collected every week (n = 3) for 12 weeks after injection (15 μmol/kg).

## Author Contributions

J.X., X.M.T., C.Y., P.L., N.Q.L., Y.L., H.B.L., D.H.C. and C.X.W.: experimental work and data analysis. L.L. and G.W.Y.: project planning, data analysis.

## Supplementary Material

Supplementary Information Ultrahigh relaxivity and safe probes of manganese oxide nanoparticles for in vivo imaging

## Figures and Tables

**Table 1:** Comparison of relaxivity of reported Mn-based NPs

Materials*	Core element	r_1_(mM^−1^s^−1^)	Field(T)	Ref.
HMnO@mSiO_2_	MnO	0.99	11.7	23
MnO@PEG-phospholipid	MnO	0.11	11.7	23
MnO@mSiO_2_	MnO	0.65	11.7	23
MnO@dSiO_2_	MnO	0.08	11.7	23
Mn_3_O_4_ nanospheres	Mn_3_O_4_	1.31	3	24
Mn_3_O_4_ nanoplates	Mn_3_O_4_	2.06	3	24
Mn_3_O_4_ nanocubes	Mn_3_O_4_	1.08	3	24
MnO nanoplates	MnO	5.5	3	42
Mn_3_O_4_@SiO_2_	Mn_3_O_4_	0.47	3	44
WMON	MnO	0.21	3	22
HMnO	Mn_3_O_4_	1.42	3	22
HSA-MNOP	MnO	1.97	7	43
Mn-NMOFs	MnO	4	9.4	45
MnO	MnO	0.12	3	46
Mn-MSNs	MnO(Mn_3_O_4_)	2.28	3	47
**Mn_3_O_4_**	**Mn_3_O_4_**	**8.26**	**3**	**Our results**

*Materials annotations: HMnO@mSiO_2_ –mesoporous silica coated hollow MnO or Mn_3_O_4_ nanoparticles. MnO@PEG-phospholipid: PEG-phospholipid coated MnO nanoparticles. MnO@mSiO_2_: mesoporous silica-coated MnO nanoparticles. MnO@dSiO_2_: dense silica coated MnO nanoparticles. Mn_3_O_4_@SiO_2_: silica coated Mn_3_O_4_ nanoparticles. WMON: water-dispersible manganese oxide nanoparticles. HMON: hollow manganese oxide nanoparticles. HSA@MNOP: human albumin coated manganese oxide nanoparticles. Mn-NMOFs: manganese-containing nanoscale metal-oraganic frameworks. Mn-MSNs: dispersing manganese oxide nanparticles into mesopores of mesopourous silica nanoparticles.
