# Supplementary material for: Ultrahigh relaxivity and safe probes of manganese oxide nanoparticles for in vivo imaging
Source: Sci Rep. 2013 Dec 5;3:3424. doi: 10.1038/srep03424 (PMC4070373; doi:10.1038/srep03424)
Supplement: Supplementary Information — Ultrahigh relaxivity and safe probes of manganese oxide nanoparticles for in vivo imaging [file srep03424-s1.pdf]

Supplementary Information

Ultrahigh relaxivity and safe probes of manganese oxide nanoparticles for *in vivo* imaging

J. Xiao [a, b, #], X. M. Tian [c, #], C. Yang [b], P. Liu [a], N. Q. Luo [a], Y. Liang [a], H. B. Li [a], D. H. Chen [a], C. X. Wang [a], L. Li [b, *] & G. W. Yang [a, *]

[a] *State Key Laboratory of Optoelectronic Materials and Technologies, Institute of Optoelectronic and Functional Composite Materials, Nanotechnology Research Center, School of Physics & Engineering, Sun Yat-sen University, Guangzhou 510275, Guangdong, P. R. China*

[b] *State Key Laboratory of Oncology in South China, Imaging Diagnosis and Interventional Center, Sun Yat-sen University Cancer Center, Guangzhou 510060, P. R. China*

[c] *Department of Biomedical Engineering, Guangzhou Medical University, Guangzhou 510182, P. R. China*

1. A schematic illustration of the experimental setup.

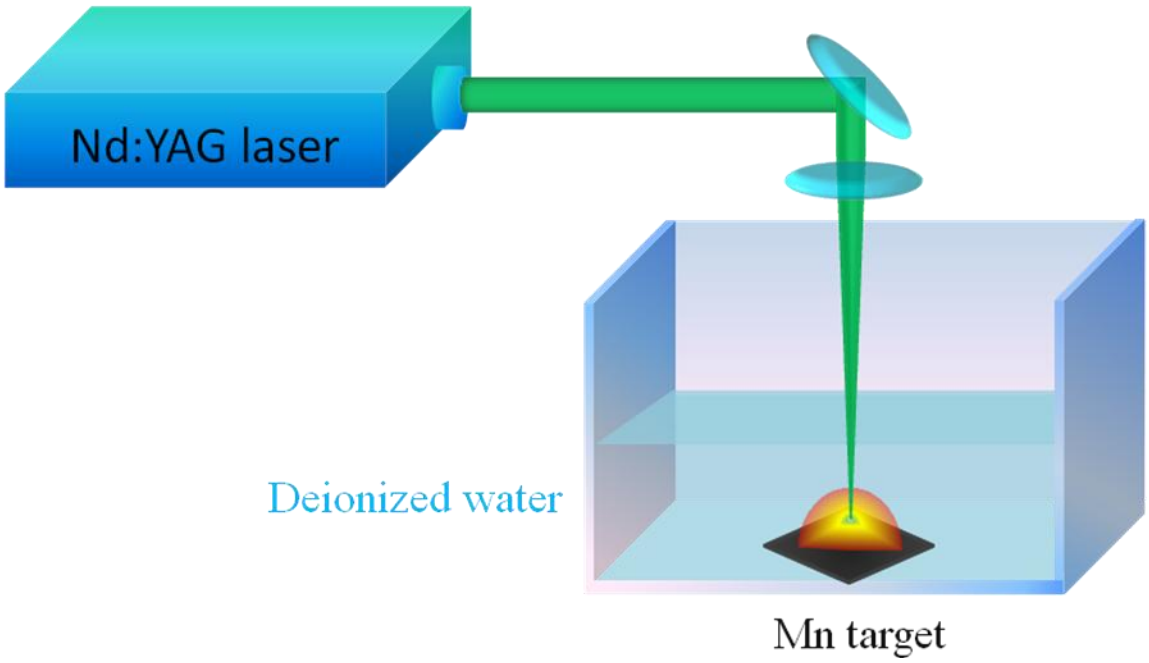

Fig. S1. Experimental setup of synthesis of Mn_3O_4 nanocrystals

2. R1 assay. The concentration of Mn was obtained by inductively coupled plasma atomic emission spectroscopy (ICP-AES, Spectro ciros vision, Spectro, Germany). Longitudinal relaxation rate ($1/T_1$, units of s^{-1}) for Mn of different concentrations is measured with inversion recovery pulse sequences with T_R=2000 ms and T_I = 500-1400 ms. T_I is fitted to an exponential T_1 recovery model by a non-linear least square regression Equation (1), from which $1/T_1$ of different Mn concentrations is determined.

$$S_{IR}(T_I) = S_0[1-2\exp(-T_I/T_1)+\exp(-T_R/T_1)] \quad (1)$$

Where T_1 is longitudinal relaxation rate, T_R is recovery time, and T_I is inversion time. S_{IR}, gray-scale values of the MR images, is provided by Picture Archiving and

Communication System (PACS) software.

Longitudinal relaxivity (r_1, units of $s^{-1}mM^{-1}$) of the Mn-NPs is calculated according to Equation (2):

$$r_1 = \Delta(1/T_1)/[Gd^{3+} concentration] \qquad (2)$$

3. The contrast experiments

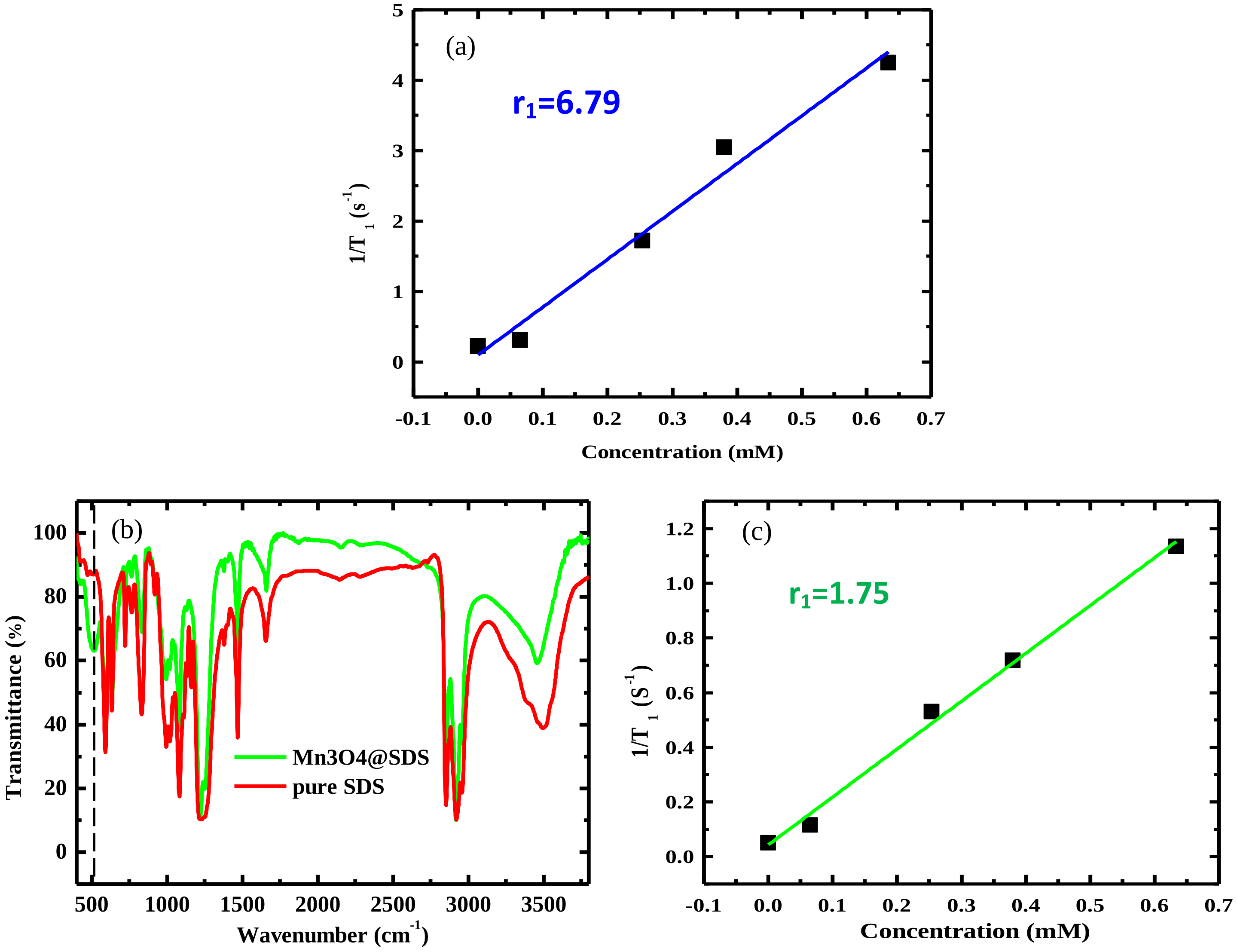

Fig. S2. (a) The relaxivity (r_1) of Mn_3O_4 NPs in PBS solution. (b) FTIR spectrum of

pure SDS and the SDS coated Mn_3O_4, The spectrum shows the characteristic absorption bands at 516cm^{-1}, which assigns as the coupling modes between the Mn-O stretching modes of tetrahedral and octahedral sites of Mn_3O_4 nanocrystals. (c) The corresponding relaxivity of Mn3O4@SDS nanocrystals.

4. Animal modal. Animal experiments were performed according to the National Institutes of Health guidelines on the rules of animal's research. Four to six weeks old Balb/c nude mice (weight of 18 to 22 g) were obtained from the animal experiment center of the Medical College, Sun Yat-sen University, China, then maintained in a specific pathogen-free (SPF) environment (Certificate No. 26-99S031). Mice were subcutaneously injected with NPC CNE-2 cells (5×10^6 in 100 μL PBS). Mice were randomized by approximately 6 mm^3 tumor after 9 days.

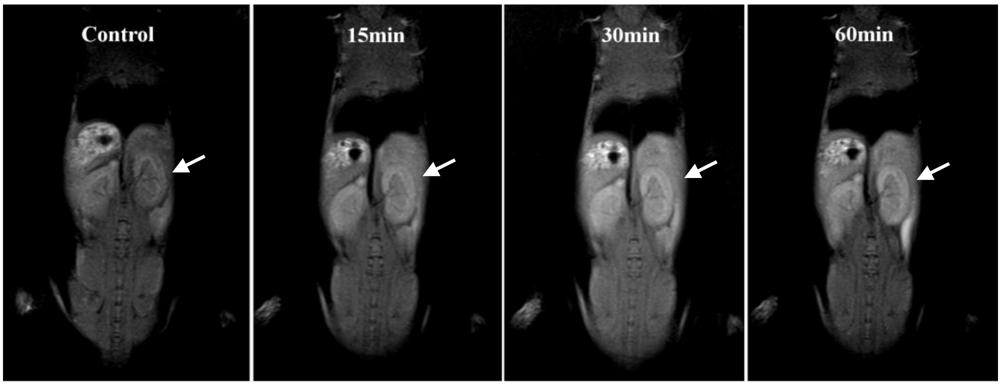

Fig. S3. Typical dynamic contrast-enhanced T1-weighted MR images of kidney (white arrow) in mice 0, 15, 30 and 60 min after intravenous administration of the Mn-NPs (15 μmol kg^{-1}).

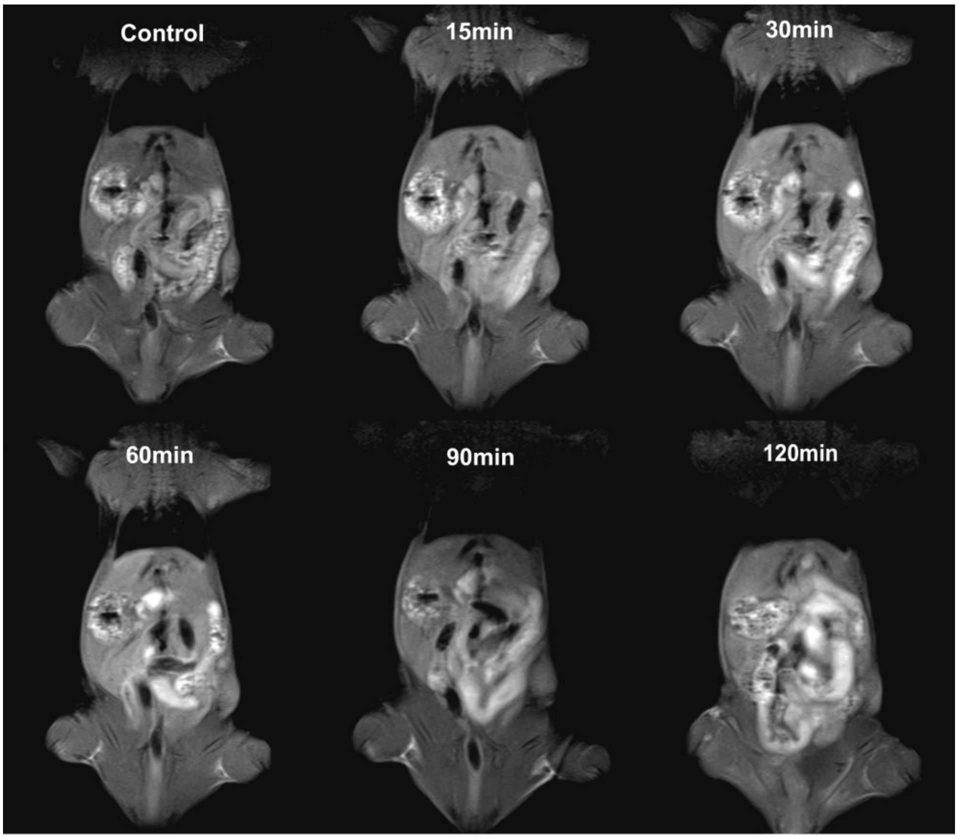

Fig. S4. Typical dynamic contrast-enhanced T1-weighted MR images of xenografted tumor in mice 0, 15, 30, 60, 90 and 120 min after intravenous administration of the Mn-NPs (15 μmol kg^{-1}).

5. The MR image after same dose injection of Gd-DTPA

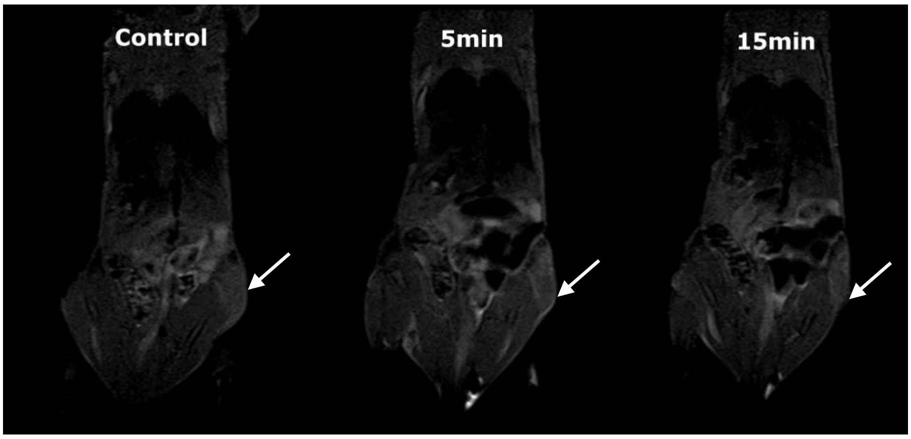

Fig. S5. Typical dynamic contrast-enhanced T1-weighted MR images of xenografted tumor in mice 0, 5, 15 min after intravenous administration of the Gd-DTPA (15 μmol

kg^{-1}). The grey values of 0, 5, 15 min are 31.5, 38.7, 35.4, respectively.

6. Cytotoxicity assay. The mouse fibrosarcoma L-929 cells in logarithmic growth period are incubated with different concentrations of the Mn_3O_4 NPs (10 μM, 1 μM, and 100 nM) in Dulbecco's modified Eagle's medium (DMEM) in 96-well plates, at 37 ℃, 5% CO_2. NP69 cells were incubated in keratinocyte-serum free medium (K-SFM , Gibco), including 25μg/ml bovine pituitary extract (BPE), 0.1 ng/ml epidermal growth factor (EGF).

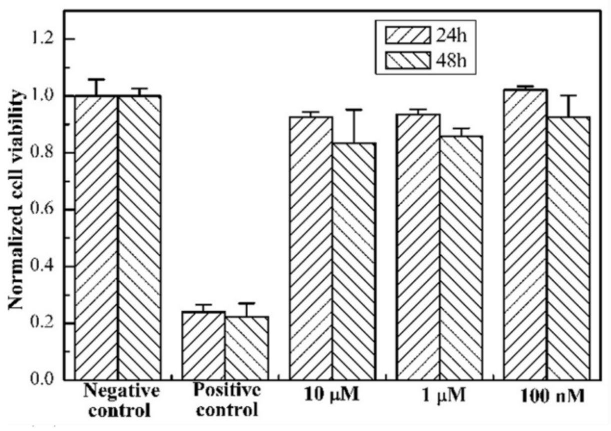

Fig. S6. The data of cell viability on L929 cells incubated with different concentrations (10 μM, 1 μM, and 100 nM) of the Mn_3O_4 NPs for 24 and 48 h.

7. Apoptosis assay. The murine macrophage line Raw264.7 cells in 6-well plants are incubated with PBS (negative control), LPS (positive control) and the Mn_3O_4 NPs (100 and 500 nM) for 48 h.

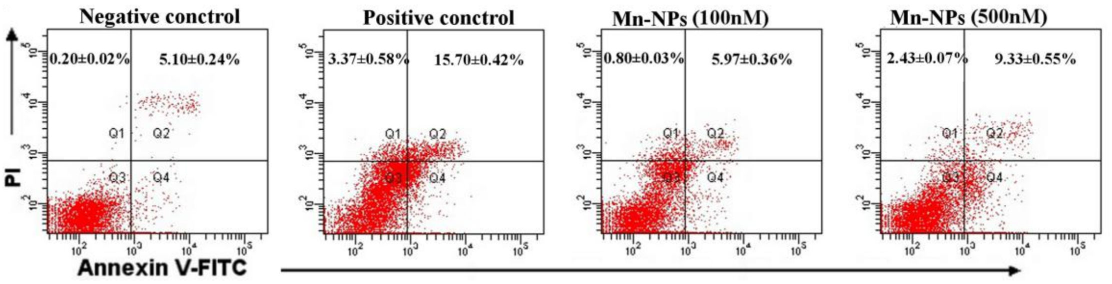

Fig. S7. Apoptosis rate of RAW264.7 cells were measured by flow cytometry at 48 h after incubation of PBS, LPS, Mn_3O_4 NPs (100nM, 500 nM). Cells were stained by annexin V and PI.

8. Absorption assay. Nasopharyngeal carcinoma (NPC) CNE-2 cells and NP69 cells were cultured in PRMI-1640 media (Invitrogen, Carlsbad, CA) with 10% fetal bovine serum (FBS, Invitrogen), at 37℃ and 5% CO_2. Cells at logarithmic growth phase were incubated with the Mn-NPs (100 μmol L^{-1}), the culture medium was removed at 12 h, fixed by 4% paraformaldehyde and 0.5% glutaraldehyde for 15 min, centrifuged at 3000 g for 15 min at room temperature. Absorption of cells was measured by using a 100-CX TEM (JEOL, Inc., Japan) at 120kV.

9. Immunotoxicity assays of the Mn-NPs

Expressions of representative cytokines of monocytes/macrophages in peripheral blood

Twenty μL of peripheral blood was collected into a tube supplemented with heparin sodium (4 μL) by vena ophthalmica. Cells were stained with anti-mouse CD206-PE, anti-mouse CD11b-FITC, anti-mouse CD80-PE, anti-mouse CD86-PE in dark for 30 min, 2 ml of erythrocytes were lysed in dark for 2 min, followed by the addition of 2

ml PBS, subsequently centrifuged at 1200 g for 5 min at room temperature, followed by the addition of 400 μL PBS. Samples were measured by flow cytometry, respectively.

Expression on CD69 of lymphocyte cells in peripheral blood

Peripheral blood (20 μL) of mice was stained with anti-mouse CD69-FITC and Anti-mouse CD3-PE in dark for 30 min, Erythrocytes were lysed (2 ml) in dark for 2 min, then added 2 ml PBS, centrifuged at 1200 g for 5 min at room temperature, followed by the addition of 400 μL PBS. Samples were measured by flow cytometry.

Expression of CD69 in peripheral lymphocytes

Mice were sacrificed by devapitation, and lymphocyte cells were isolated from subclavicular, grounding submandibular, axillary, superficial inguinal and mesenteric lymph nodes, and obtained through 200 meshs under sterile conditions. Samples were then centrifuged at 300 g for 5 min at room temperature, and incubated with anti-mouse CD69-FITC and anti-mouse CD3-PE in dark for 30 min. Samples were measured by flow cytometry.

Statistical analyses

All data are expressed as means ± SEM, statistical analyses were performed by one-way ANOVA with *t* test by using origin 8.0 software.

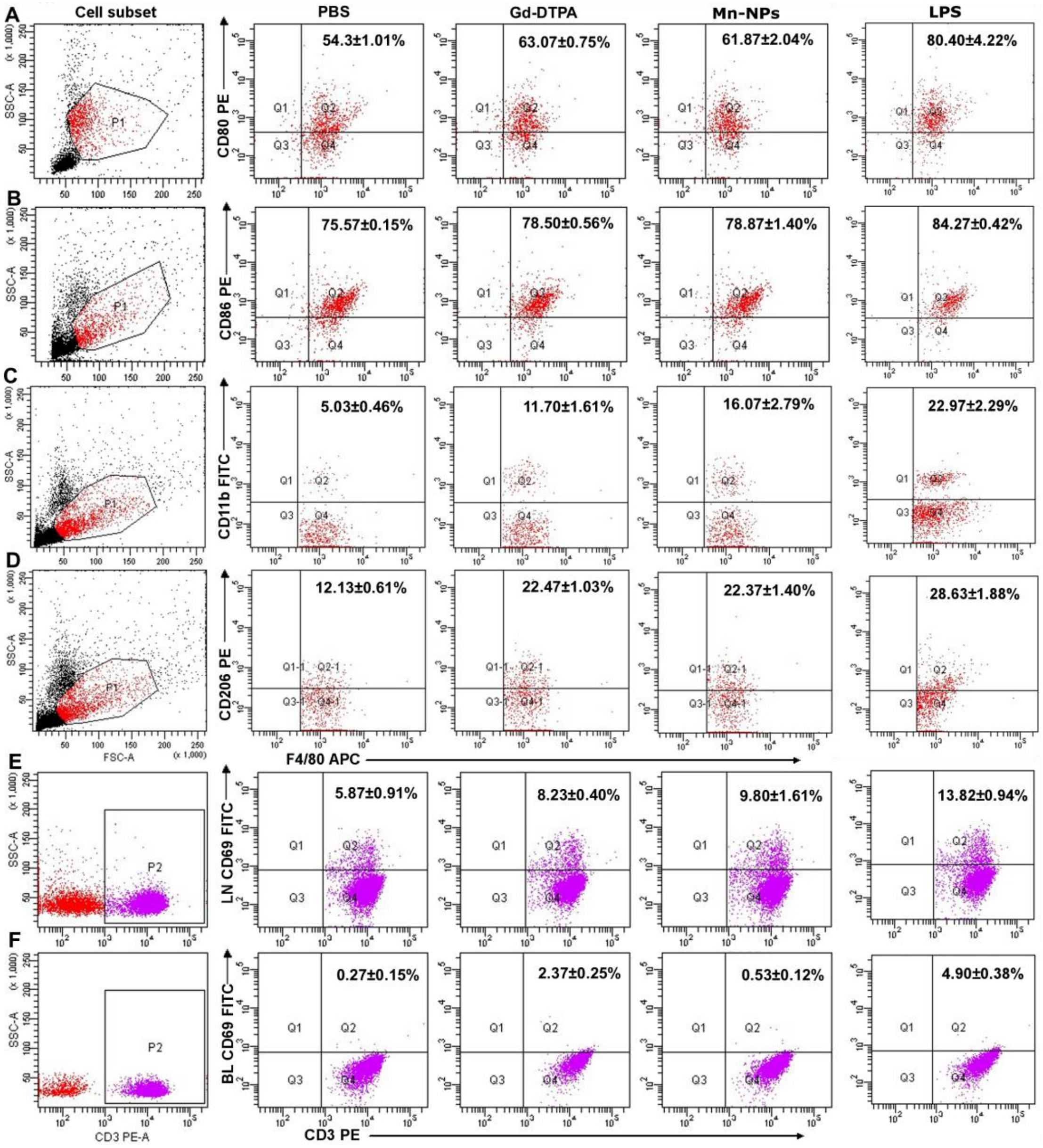

Fig. S8. Histogram plot of immunotoxicity assay. CD80, CD86, CD11b and CD206 expression of monocytes/macrophages in peripheral blood; as well as CD69 cytokine of adaptive immune in lymphocyte cells of peripheral blood (BL) and lymph nodes (LN).

10. Inductively Coupled Plasma Mass Spectrometry Assay. A Thermo Electron X7, ICP-MS (Thermo Instrument System Inc. America) was used for assay. Blood (5-10 μL) or tissues was collected at each time point, samples were weighed prior to the addition of 5 ml nitric acid (HNO3, Merk) heated by an electric stove. After complete

digestion and cooling, 2% HNO3 was added to 25 ml for ICP-MS analysis.

11. Histochemistry. The tissues including brain, heart, kidney, liver, lung and spleen were collected at 4 h after injecting the Mn-NPs (15 μmol kg^{-1}), the samples were soaked into 4% paraform immediately for 24 h, then embedded in paraffin, sectioned, and stained with H&E and Prussian Blue.

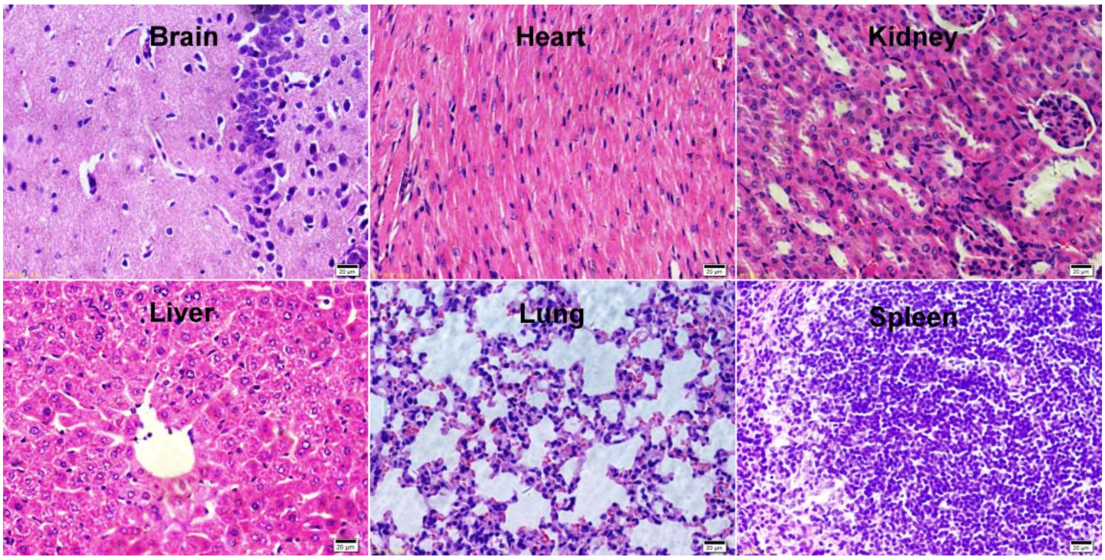

Fig. S9. Typical tissues were collected immediately after injection of the Mn-NPs (15 μmol kg^{-1}), stained with HE.

Table S1. Concentrations of Mn (ng/g) measured by ICP-MS in various organs of our mice model (n=3) at 4, 10 and 24 h after intravenous injection of the nanoprobes (15 μmol kg^{-1}).

Mn (ng/g, mean ±S.E)	4 h	10 h	24 h
Brain	2445.88 ±57.46	1403.63 ±38.05	556.62 ±21.45
Liver	5342.99 ±97.54	27180.66 ±1029.51	51591.29 ±479.31
Lung	36000 ±2858.65	25941.07 ±759.39	9288.83 ±880.72
Spleen	4096.2 ±245.06	10594.2 ±1186.46	26806.93 ±908.6
Heart	324.67 ±15.02	803.71 ±20.04	1077.22 ±34.36
Kidney	542.26 ±30.57	1296.25 ±36.22	1736.72 ±20.73
Tumor	708.75 ±25.92	3334.1 ±85.7	5618.75 ±134.98
